# Trauma and Activism: Using a Postcolonial Feminist Lens to Understand the Experiences of Service Providers Who Support Racialized Immigrant Women’s Mental Health and Wellbeing

**DOI:** 10.3390/ijerph22081229

**Published:** 2025-08-07

**Authors:** Judith A. MacDonnell, Mahdieh Dastjerdi, Nimo Bokore, Wangari Tharao

**Affiliations:** 1 School of Nursing, York University, 3rd Floor HNES Building, 4700 Keele Street, Toronto, ON M3J 1P3, Canada; dastjerd@yorku.ca; 2 School of Social Work, Carleton University, 1125 Colonel by Dr., Dunton Tower, #618, Ottawa, ON K1S 5B6, Canada; nimo.bokore@carleton.ca; 3 Women’s Health in Women’s Hands Community Health Centre, 2 Carleton Street, Suite 500, Toronto, ON M5B 1J3, Canada; wangari@whiwh.com

**Keywords:** racialized, immigrant women, mental health promotion, activism, trauma, service providers

## Abstract

The global Black Lives Matter movement and COVID-19 pandemic drew attention to the urgency of addressing entrenched structural dynamics such as racialization, gender, and colonization shaping health inequities for diverse racialized people. Canadian community-based research with racialized immigrant women recognized the need to enhance service provider capacity using a strengths-based activism approach to support client health and wellbeing. In this study, we aimed to understand the impacts of this mental health promotion practice on service providers and strategies to support them. Through purposeful convenience sampling, three focus groups were completed with 19 service providers working in settlement and mental health services in Toronto, Canada. Participants represented varied ethnicities and work experiences; most self-identified as female and racialized, with experiences living as immigrant women in Canada. Postcolonial feminist and critical mental health promotion analysis illuminated organizational and structural dynamics contributing to burnout and vicarious trauma that necessitate a focus on trauma- and violence-informed care. Transformative narratives reflected service provider resilience and activism, which aligned with and challenged mainstream biomedical approaches to mental health promotion. Implications include employing a postcolonial feminist lens to identify meaningful and comprehensive anti-oppression strategies that take colonialism, racialization, gender, and ableism and their intersections into account to decolonize nursing practices. Promoting health equity for diverse racialized women necessitates focused attention and multilevel anti-oppression strategies aligned with critical mental health promotion practices.

## 1. Introduction

The global Black Lives Matter movement and the COVID-19 pandemic have drawn attention to the urgency of addressing deeply entrenched structural and social dynamics. Racialization and gender and their intersections, in the larger context of colonization, for example, contribute to health inequities that limit the health of diversely situated racialized groups, e.g., [1,2,3,4,5,6,7,8,9,10,11,12]. A solid body of research shows that racialized immigrant and refugee women can face a range of mental health and wellbeing (MHW) challenges during the migration and settlement process. These socially and structurally determined challenges include forms of systemic violence such as social norms or public policies resulting in unemployment or underemployment, social exclusion, discrimination and stigma impacting their wellbeing. Known as structural violence, these “indirect forms of violence… are built into social structures and… prevent people from meeting their basic needs or fulfilling their potential” [13] (p. 40). For the past decade, studies have shown that racialized immigrant women, despite these challenges, also show strength and resilience that help them transform their communities and systems of care to be more responsive to their needs, e.g., [9,14,15,16].

However, most research on the MHW of racialized immigrant and refugee women addresses access barriers and experiences of care, with less attention paid to the experiences of health and/or social service providers (SPs) involved in their care. Nursing researchers have engaged SPs as key informants to understand barriers to client access [3,17,18,19]. Interdisciplinary researchers in health, nursing, social work and psychology have undertaken research for/with diverse groups of SPs to address their experiences and needs for support [1,13,15,16,19,20,21,22,23]. Over the last two decades, and with the global movements and COVID-19, structural dynamics such as deeply embedded racialization and colonialism have contributed to the vulnerability of racialized communities resulting in health inequities. Canadian critical health, social science and nursing researchers have applied critical and community-based methodologies to examine how complex dynamics of power and privilege, in a colonial context, intersect to shape the lived experiences of diverse racialized communities and the strategies needed to improve their everyday lives. This includes those including those who are SPs, those who self-identify as sexual and gender diverse people and those living with disabilities, e.g., [1,2,15,22,23,24,25,26,27,28].

It may be useful to explain several concepts related to critical and community-based methodologies that we include in this paper. Critical methodologies take into account how complex dynamics of power and privilege are embedded in social norms such as racism that normalize privilege for certain groups (e.g., White groups) over others (e.g., racialized groups) with a view to addressing actions for change aligned with social justice goals [25]. We apply a definition of racialization as explained by the Canadian Research Institute for the Advancement of Women (CRIAW) [29] (p. 2): Individuals and groups “who experience[] racism because of their race, skin color, ethnic background, accent, culture, or religion” [29] (p. 2). Although they embody many differences, “what they have in common is that they are racialized [and seen as the Other]; they are subject to racism and made to feel different because of their racial/ethnic/background” [29] (p. 2). Contemporary critical feminist methodologies foreground gender as a key dynamic and often account for intersectionality. An intersectional approach takes multiple factors into account in order to understand the complexity of our worlds such as in relation to social inequality in a given society [30]. As Hill Collins and Bilge [31] explain, “People’s lives and the organization of power in a given society are better understood as being shaped not by a single axis of social division, be it race, gender, or class, but by many axes that work together and influence each other” [31] (p. 2), illustrating unique experiences of privilege and oppression. Power is often conceptualized as oppressive, as “power over” but it can also be considered in its positive form as “power to” act such as exercising one’s agency to resist oppressive structural dynamics which are deeply embedded in society such as stereotypes, discrimination and stigma which create barriers to accessing care [29]. Postcolonial feminist approaches [25,30,32,33], account for systems of domination based on gendering and racialization such as within the health care system. As Montague et al. [34] note, “anti-Black and anti-Indigenous racism have their origins in European colonialism through the enslavement of Africans and the displacement and marginalization of Indigenous peoples” [34] (p. 113) which also involved economic exploitation [30]. The deeply embedded Eurocentric Western ideology of “racial superiority…, [historically] used to justify oppression, marginalization and the dehumanization of these communities” [34] (p. 113), also constructed racialized people as “the Other.” Such dynamics continue today, erasing and diminishing their knowledges and practices…” with ongoing impacts of oppression, discrimination and racism” [32,34] (p. 113). Phrases such as “post-colonial” and “anti-colonial” approaches represent various strategies of resistance to colonialism and its impacts in contemporary society [30].

Community-based research (CBR) attends to the complexity of power and privilege in research, aiming to foster meaningful community–academic collaborations that centre community voices across all phases of the research, reflecting priorities on building community strengths and enhancing community capacity [24]. Earlier CBR [24] with racialized immigrant women centred the voices of these communities in relation to how racialized immigrant women express their agency individually and collectively to push against systems for transformative change. The findings challenged dominant notions of political activism which are often equated with collective action in the public sphere (e.g., mass protests). Instead, findings based in critical research [24] informed by a postcolonial feminist lens, prioritizing the subjectivities and experiences of racialized immigrant women, suggest that the meanings of their everyday activism are shaped by gender, racialization and migrant status as well as notions of what it means to be a good citizen. In this context of Western and Eurocentric domination, individual activism includes actions that resist dominant discourses that marginalize or silence racialized immigrant women or dismiss their knowledges and contributions to society. Thus, forms of activism include, but are not limited to forums for collective dialogue or mass protests, dancing, cooking and “storytelling, which make[s] visible everyday subjugated knowledges, emotions and actions” [24] (p. 13) that are informing their action for transformative change.

Critical research has been undertaken with racialized women SPs who work in female-dominated health services fields such as mental health nursing, settlement and gender-based violence, e.g., [13,35,36,37,38]. Such research explicitly accounts for complex structural dynamics including gender, racialization, colonialism, and neoliberal influences that shape SP worklives where the historical context of women’s activism was integral to creating organizations that could serve racialized women’s needs in the first place [35,37,38]. As Singh [36] notes, ethno-specific anti-violence agencies for women were a response by activists to address the institutionalized racism in mainstream organizations, as well as both “government and grassroot initiatives where racialized migrants were deemed optimal in addressing gender violence in their own communities” [36] (p. 511). Settlement and mental health sectors have historically been devalued compared to other sectors [13,38,39]. In addition, neoliberal dynamics of efficiency and cost cutting measures have constrained health system and organizational level SP resources, while the gendered colonial and racialized structures that SPs, especially those with experiences as migrants in these sectors can face, is highly relevant. In this research SPs identified and problematized bureaucratic constraints and a focus on efficiencies, such as in Toronto-based organizations that serve immigrants as contributing to SP moral distress [28,36]. SPs at times engaged in activism to challenge such concerns within their agencies and beyond to address the system level issues that contributed to the health inequities their clients and communities faced [28,36,37].

There are psychological and emotional risks for SPs working with immigrant and refugee women survivors of trauma. As Knight [40] and Varcoe [41] note, SP risk for secondary traumatic stress, vicarious trauma or compassion fatigue, forms of indirect trauma, can be high. A review of related literature, e.g., [42,43] suggests that a self-care discourse for SPs often prevails, whereby organizations mainly promote employee MHW with attention to their own coping skills. To a lesser extent, this literature has focused on structural and organizational underpinnings of SPs’ practice that contribute to SP trauma and burnout [28,41,43,44,45,46]. Until just recently, limited literature on trauma-informed care has explicitly mentioned structural dynamics of gender and racialization in relation to creating a supportive organizational climate for SPs. With the onset of the COVID-19 pandemic, a burgeoning body of new research emerged on SP-related moral distress, trauma and burnout, e.g., [47,48]. In a subset of that literature, some critical researchers pointed out the existence of structural inequities shaping workplaces and contributing to pandemic-specific factors increasing work-related stress and burnout [4,6,7,8,10,28,48,49,50].

There is a body of critical MWH research that characterizes understandings of and practices to support MHW that departs from those reflecting prevailing biomedical and behavioural discourses as forms of structural violence. A range of researchers highlight the constructedness of Western mental health knowledge, practice and systems based in dominant biopsychiatry as mechanisms of social control. Yet, they problematize the invisibility of the global and historical links to colonization processes that create and maintain racial and gendered inequities for diverse groups whose MHW is always subject to normative and Western professional knowledge and authority [39,51,52,53,54,55,56,57,58,59]. Racine [32] foregrounds “subjugated knowledge,” noting the value of the postcolonial approach in “uncovering the exclusionary effects of dominant ideologies in ‘Othering’ other forms of knowledge” (p. 95). Gailits et al. [59], whose recent research with Latin American, female immigrants emphasizes the epistemic violence they face, noting their experiences of migratory distress must be interpreted beyond biomedical understandings, given the larger context of their lives which have been shaped by colonization and racialization. Similarly, Morrow and Malcoe [39] consider critical MHW perspectives, which are silenced and marginalized, forms of epistemic violence.

In this vein, critical research methodologies, such as postcolonial feminism, that affirm the importance of intersectionality and centering racialization and migrant status along with gender can illustrate how subjugated knowledges and practices, which are often invisible or have been marginalized in mainstream North American health systems, are relevant in the care of racialized immigrant women and the SPs working with them [4,9,28,30,31,32,33,34]. A critical approach to mental health promotion is relevant as described by Morrow and Malcoe [39] who lay out the way this approach challenges the dominant biomedical perspective that permeates MHW promotion and health care and which itself contributes to health inequities for equity-seeking groups such as racialized people. According to Morrow and Malcoe [39], “the official story of mental health being told by biomedicine increasingly claims that all forms of emotional suffering are ‘disorders’ and that ‘mental illness’ is a major contributor to the total global burden of disability and disease” [39] (p. 6). To counter the claims of biomedicine’s hegemonic hold on prevailing professional perspectives that shape societal understandings of effective MHW practices that align with psychiatric and pharmaceutical treatment and support, they call for largely absent “perspectives and forms of evidence that start with an analysis of power and consider the social, political, cultural, and economic production of mental health problems and solutions” [39] (p. 6). Integral to a critical mental health lens is making visible “diverse voices of experience-psychiatric survivors and others who have lived with various forms of social marginalization and emotional suffering” [39] (p. 6).

Canadian CBR [24] with racialized immigrant women recognized the need to enhance SP capacity using a strengths-based, activism approach to support client MWH. We noted that no research had addressed SP needs in relation to activism-based MHW promotion [9]. We thus undertook this focus group (FG) CBR study aiming to understand SP needs in relation to their use of strengths-based approaches such as activism to promote the MHW of racialized immigrant women *clients*. We applied postcolonial feminist and critical MHW lenses [32,39] to explore activism-based MHW resources geared to SPs that they could apply to support their clients’ MHW. However, unexpectedly, two themes emerging from FG analysis pointed to the impacts of this MHW work *on SPs themselves*.

As noted in the above reviewed literature, recent critical research identifies the importance of violence- and trauma-informed workplaces to support the MHW of SPs and indicates there are MHW risks for SPs who work with immigrant and refugee survivors of trauma. To our knowledge, however, there is no critical research that examines the MHW of SPs in relation to their MHW promotion work with racialized immigrant women using strengths-based approaches such as activism. Our research thus responds to this gap in a unique way with a goal of supporting SP in their MHW practice. Our research question is: “What are the impacts of this MHW practice using activism- based strategies with racialized immigrant women on Canadian mental health and settlement SPs and what are the implications for supporting them in their practice?” We apply a broad definition of racialized immigrant women and a definition of activism that is grounded in racialized immigrant women’s subjectivities and experiences in relation to SP vicarious trauma and burnout and SP activism.

In this paper, we discuss these two themes that relate to the personal and professional impacts of this work on SPs themselves: *Burnout and Vicarious Trauma* and *Breaking the Silence: SP Activism*. We foreground systemic barriers, personal and professional impacts on SPs and strategies to support their MHW. We then discuss the implications of this research, including recommendations to promote the MHW of diverse racialized immigrant women SPs.

## 2. Materials and Method

### 2.1. Methodology and Procedures

Using a CBR approach and qualitative design, this study centred the histories and voices of members of the communities themselves, aiming to build community capacity and attending to complex power dynamics to understand the unique needs of SPs who support the MHW of racialized immigrant women. The study focused on creating meaningful resources for SP MHW practices. We utilized purposeful convenience sampling, paying attention to the need for researcher reflexivity and the complex dynamics of power and privilege, including race and gender that shape the meaning-making process of SPs [30,32]. As Racine [32] stresses, a postcolonial feminist lens provides the analytic lens to look at the impact of these factors in shaping health experiences with attention to the “dominant health ideologies that underpin [SPs’] everyday practice and the structural barriers that may constrain the utilization of public healthcare services by non-Western populations…” (p. 91). Our approach to postcolonial feminism, as outlined by Racine [32] and Collins [30] is informed by scholarship rooted in diverse critical feminist, anti-racist, postcolonial, and intersectional methodologies as extensively examined by Collins [30].

Approval was obtained from the institutional Research Ethics Board prior to recruitment. The researchers explained the study process to each participant emphasizing confidentiality and the informed consent process, which included the right to opt-out at any time without any negative consequences. Written informed consent was obtained from each participant to take part in one in-person audiotaped FG. Participants were asked to keep the information shared in the FGs confidential. Each participant also completed a brief demographic form. Identifying information about individuals and agencies was removed after transcription to protect privacy and confidentiality; none was reported in findings. Data was secured and retained as per the principal investigators’ institutional policy.

SPs participated in one of three in-person FGs to discuss their understandings of activism and their strategies to support the MHW of racialized immigrant women clients in their care. Each of these three in-person and audiotaped FGs was conducted with a separate set of SP participants. We sought their feedback on the usefulness of several activism-based project resources for SPs. We used semi-structured interview guides to explore SP understanding of activism and the barriers they face in promoting client MHW.

### 2.2. Participants

Three FGs were completed in 2017 with 19 mental health and settlement providers with diverse levels of education, age and work experience in the GTA. Most participants self-identified as female, racialized, and immigrants living in Canada. See Table 1 for detail about the participant sample. One of the 19 participants self-identified as gender-fluid; as such, in this paper we use the terms they/them as we represent the participant voices.

### 2.3. Analysis

We applied a postcolonial feminist lens with attention to how complex dynamics of power related to intersections of gender, colonialism, and racialization, for instance, shaped the everyday lives of participants and the structures of their work and informed understandings of mental health and mental health promotion. We also applied a critical mental health promotion lens, drawing from Morrow and Malcoe [39]. Transcripts were thematically analyzed manually using a critical postcolonial feminist lens that foregrounds racialization [32] to identify codes which contributed to subthemes under broader themes. Data collection and analysis were conducted concurrently. There were copious codes under each subtheme and as noted in the table, several codes were common to more than one subtheme. To illustrate the thematic structure, examples of subthemes and codes that were relevant to the first main theme, Vicarious Trauma and Burnout are reflected in Supplementary Materials: Table S1: Thematic Structure: Example from Selected Theme, Subthemes and Codes.

The research team met regularly to consider how power and privilege, such as gendered and racialized dynamics, shaped themes such as SPs’ experiences of activism to challenge systems to be more responsive to their clients’ needs. Initial themes emerging from the first two FGs were shared in the 3rd FG to stimulate discussion (e.g., barriers faced by SPs in their practice).

Through ongoing analysis and reflection, our team identified themes that foregrounded SPs’ experiences and dynamics of power that shape their ability to promote client MHW and ways that SPs themselves participate in activism to make the system more responsive to their needs. We took steps to ensure rigour and trustworthiness [60], such as member checking in a forum geared to policy, program, research and community stakeholders where we shared study findings in a panel discussion with breakout sessions where we often heard that the findings resonated with diverse stakeholders [61]. We also shared a lay information summary for these stakeholders on SP activism and vicarious trauma with diverse audiences in conference presentations over the next two years [9,24,61]. Post-COVID, we began to review our data from different perspectives, influenced by an emerging body of critical mental health literature addressing structural violence, colonization and trauma-and violence informed care, e.g., [28,35], as well as dialogue on decolonizing nursing post-COVID-19, e.g., [25,51,56,62]; this paper reflects that engagement. Reflexivity was a key dynamic as the researchers considered their positionality relative to power and privilege and its implications across the study process [32,63]. Our team, diverse with respect to racialized and immigrant positionalities, education and lived experience and representing expertise in nursing, social work, community and public health, offered both insider and outsider perspectives. We brought significant experience in critical methodologies and qualitative CBR with racialized immigrant women communities and expertise in trauma and the mental health and settlement sectors.

## 3. Results

SPs described a range of MHW promotion strategies including self-care as a method of survival through storytelling about their shared values and motivation for providing services. SPs use similar strategies to improve their clients’ capacity and to encourage them to advocate for themselves for their rights and to make a change. SPs indicated that while they were committed to their work, organizational level support was often missing. SPs shared the impact that limited structural or organization resources and support systems have on their MHW, focusing on the following two areas: The first was the impacts of vicarious trauma and workplace burnout on SPs; and the second, on how they use their voices and actions to break the existing silences around the MHW impacts of this practice on SPs through activism.

### 3.1. Vicarious Trauma and Burnout

SP narratives illuminated multiple and interwoven factors related to their carework of promoting the MHW of their racialized immigrant and refugee women clients that contributed to this first theme of vicarious trauma and burnout. SPs described three factors (subthemes identified here) that contributed to vicarious trauma and burnout and the personal and professional impacts on them: (1) constrained client support systems; (2) sector-specific training and organizational mandates; along with (3) the relevance of racialized immigrant women SPs’ experiences of migration and settlement to their everyday MHW work. As SPs shared the complexity of individual (micro), organizational (meso) and system (macro) level dynamics that contributed to their lived experiences of burnout and vicarious trauma, a fourth subtheme emerged: (4) SP insights into strategies to support SP MHW.

#### 3.1.1. Constrained Client Support Systems

A key factor that contributed to SP burnout and vicarious trauma was the current landscape of constrained client support systems that were linked to other multilevel and interwoven factors such as the availability of client resources and programs, as well as the increasing number of clients with complex needs. In these narratives, it was clear that client support systems were affected by macro-level immigration and resettlement policies as well the availability and accessibility of sector- and agency-specific resources and programs (meso-level) geared to diverse clients whose lives are shaped by social and structural determinants of health such as poverty, lack of housing and discrimination. SPs were constantly challenged to keep tabs on current policies, programs and resources in an environment where these were often subject to change with little notice. As SPs spoke of the meaningful work of MHW practice, they also shared that this work has impacts on their own MHW, often describing experiences of emotional upheaval and feeling overwhelmed by heavy caseloads and burnout. As one SP shared, “It’s getting harder and harder to really know anything about anything… everything is changing before us… constantly… I think that’s where a lot of the burnout comes from… you’re supposed to be a generalist… information overload from everywhere” (FG2). Although SPs wrestled with these ongoing changes, they highlighted the importance of their relational skills and commitments to meeting clients “where they are” and thus supporting client MHW to the extent possible. As a second SP came to realize, they did not need to have all the answers when clients saw them as a sounding board: “I just need you to just listen so that I can process all this chaos that’s in my head” (FG2). Despite the barriers, this settlement SP offered an example of how they do support client MHW needs in the moment, when clients say, “Can you just put it down on paper? Can we just kind of look through it and talk about it? You don’t have to offer me solutions” (FG2). More often than not, however, SP narratives stressed that given the constrained systems they were often distressed by their limited ability to effectively serve the increasing numbers of clients who were experiencing significant and multiple issues ranging from food insecurity to housing and family violence which also contributed to SP burnout and moral distress. As several SPs stressed, with half-hour client appointments scheduled, “[It] is frustrating, like I could not give you complete attention because another client is waiting, right?” (FG3). As a FG1 SP shared, their clients also expressed concern about uneven and inaccessible resources for immigrants, saying, “I feel guilty… horrible because … I explain funding and how that works, but it does not free them to feel better. They hear facts, but it does not explain why they are being treated so vastly different in this country” (FG1). It was evident that structural issues, both organizational (meso-level) factors (e.g., client scheduling) and system (macro-level) factors (constrained resources, uneven programs, frequently changing policies and funding as well as social determinants of health such as housing) contributed to MHW challenges of burnout and moral distress.

#### 3.1.2. Sector-Specific Training and Organizational Mandates

Sector-specific SP training and organizational mandates were also factors that contributed to SP burnout and vicarious trauma that SPs predominantly discussed in relation to settlement SPs. The settlement sector is staffed by SPs who bring expertise to support their organizational mandate focus on resettlement and integration. The mental health sector is explicitly focused on supporting client MHW in a way that has often prioritized clinical diagnosis and treatment of clients including those with experiences of trauma; SPs bring mental health clinical credentials and expertise. Settlement services are often a place where clients feel safe to disclose trauma and MHW issues; they often share life stories, feeling safe without fearing stigma or judgement often attached to mental health. According to the SPs, the two sectors, however, are not well connected, and hence, silos between the mental health and settlement sectors were identified as contributing to the challenges that SPs in both sectors face. Mental health SPs often have limited knowledge of migration and resettlement policies and programs. However, SP narratives mainly focused on challenges faced by settlement SPs who often have limited mental health training and/or connections to MHW resources that are often available to SPs working in the mental health sector. As one SP from the settlement sector noted, despite increased awareness about the relevance of housing and employment to mental health, services are lacking: “… we… struggle a lot to offer adequate support… we are not clinicians…. When people disclose and are unwilling to disclose the same stories to a family doctor or mental health community agencies, we are the only first responders they talk to” (FG3). As another settlement SP shared, “We are very accessible… approachable. They do not wait 6 months to see us… I cannot get prepared. They come with a story… how they are looking for employment or language training, but they start talking about almost everything they experience” (FG3). In recent years, in the settlement sector, in particular, there is significant increase in the number of clients who disclose mental health issues or who are in crisis. At the same time, SPs must meet organizational targets and work within narrow agency constraints, such as funding mandates that limit the focus of their interaction where one in three clients disclose mental health crises or suicidal thoughts (FG3). As one SP remarked, “… we can only work with this client to do this and this” (FG1). Another SP shared the challenges of offering relevant services “if they’re still in denial” (FG3), noting that more than several visits to even an experienced settlement worker may be needed.

#### 3.1.3. The Relevance of Racialized Immigrant Women SPs’ Experiences of Migration and Settlement to Their Everyday MHW Work

Vicarious trauma was also linked to racialized immigrant women SPs’ own experiences of migration and racialization. Although SPs needed to feel that they had a safe space to engage with their clients in relation to MHW, their narratives suggested that this did not reflect their experience; neither did they identify MHW resources that addressed the relevance of SPs’ experiences of migration and settlement to their practice. We did not ask SP participants whether they were racialized immigrant women themselves, yet many SPs in the FGs spoke to the relevance of this personal experience to their journey in becoming SPs and its relevance to meeting the needs of their clients. Client stories about injustice related to gender dynamics and racism, for example, as well as client stories of settlement challenges often resonated with SPs’ own lived experiences of facing everyday structural dynamics of stigma and discrimination related to colonization and racialization, for instance. They were often like “first responders” to a number of clients who were not only sharing their current settlement concerns such as finding housing, but at times for the first time, often deeply disturbing details of traumatic migrant experiences from their homelands, in shelters, and refugee camps, for example. The embodied nature of hearing these stories from clients repeatedly during their everyday work creates emotional exhaustion especially since many of the SPs themselves had experienced it. Although they did not describe it as such, these SPs faced vicarious trauma as they listened to complex and painful stories shared by clients and in a context often of having difficult lived experiences of discrimination that can surface as they provide support for their immigrant and refugee clients. Although SPs’ individual stories point to individual SP’s personal risk for trauma that would be considered a micro level factor contributing to vicarious trauma, for which SPs would identify (micro level) personal coping strategies, when taken collectively, the structural nature of these experiences becomes clear, with implications for organizational (meso) and sector (macro level) support. SPs described experiencing tensions from hearing stories from people traumatized by violent and painful life experiences including from war-torn countries. A SP in FG1, reflected, “There’s a lot of pain. People look at you with very painful expressions that… [as one shared]… I’m coming from Darfur. I’ve seen things that no human should ever see” (FG1). Another SP offered an example of a client from Nigeria who spoke to “a lot trauma, a lot of very terrible things, maybe as bad or compared to the [names people from a specific region], and they said, ‘Why not me?”’ (FG1). They were distressed that settlement programs and resources did not seem fairly distributed. “In my case, what I answer is, ’I’m an immigrant too… I completely agree. It’s not fair. It’s not right’” (FG1). SPs often feel stuck and lack time and resources to address their own need for supports, identifying organizational barriers that they face such as feeling isolated without a place to share these feelings and concerns. As one shared, “across the board, all of us are overworked…. I am going from Rexdale to Scarborough [across the city] in one day, you know what I mean?” (FG1). Another SP expressed the cumulative impact of their work, including being a witness to the client’s past trauma shared during the session. This SP said: “It’s too much, and people have too much to do on top of kids and mortgage and whatever else goes on in life” (FG1), increasing their level of stress and incidences of experiencing various traumas.

#### 3.1.4. SP Insights into Strategies to Support SP MHW

Discussion of the three subthemes described above relating to structural (macro), organizational (meso) and individual (micro)-level factors that contribute to SP vicarious trauma, burnout and moral distress, along with insights shared by several SPs who work in teams, have implications for developing MHW strategies to support diverse SPs—the focus of this fourth subtheme. Many SPs stressed the need for organizations to enhance settlement SP access to MHW training that address, but go beyond, individual SP coping strategies (micro level), to include MHW information and resources, as well as develop processes that enhance timely client referrals to MHW support. Those SPs working in the mental health sector recommend that their agencies foster access to training to build on their understanding of current community programs and resources, such as housing that support immigrant women. Furthermore, MHW resources and training geared to SPs in both sectors need to account for SPs’ own experiences of migration and racialization that contribute to vicarious trauma. Several SPs working in teams identified mechanisms for support available to them, such as SP peer networks that reduce SP isolation by offering them emotional and interpersonal support as well as access to timely information about current programs and resources. SP narratives suggested that such peer networks can bridge silos between the sectors, providing a space for SPs within and across sectors to share their stories and concerns with interdisciplinary colleagues. Such networks offer opportunities to build their understanding of the complex structural issues such as workplace scheduling, training time constraints, agency mandates, as well racialization, and stigma that contribute to SP MHW concerns and can inform individual or collective SP action. As such, insights, based in these SPs’ own experiences and reflecting their collective voices, point to the need for comprehensive and multilevel individual, organizational and system level strategies and involving both sectors to address SP MHW. Despite the range of suggestions that were informing SP’s perspectives on the importance of MHW support for SPs, SPs also spoke of the pressure they felt to maintain a professional stance that prevented them from speaking out to share their experiences with colleagues. This perceived silencing also magnified the moral distress that shaped their everyday work.

### 3.2. Breaking the Silence: SP Activism

In this next section, we explore the second theme, which focuses on the actions that SPs take to break that silence and their activism to address the structural barriers within the system that limits their ability to consistently and effectively support their clients with implications for their own MHW. Factors that emerged as relevant to SP activism narratives and identified as five subthemes here include: engaging community voices; multilevel transformative action; the anti-colonial approach and activism; the counternarrative of ableism, MHW, and activism; and investing in activism: weighing the personal and professional considerations.

#### 3.2.1. Engaging Community Voices

Despite the limited time or resources that SPs often have available to them to address these structural and social dynamics that they identified as having significant impacts on their own MHW, many did take action that they identified as forms of activism. One of the priorities that emerged from SP narratives was a focus on centring community voices, representing their issues in development of the meaningful client or community support. Firstly, several SPs conveyed taking steps supporting their MHW to fulfill their commitment to serving communities and avoiding secondary or vicarious trauma. This included using their support systems to prepare themselves to hear the diverse voices of their clients’ communities. This individual motivation allowed SPs to meaningfully respond to issues clients raised in their one-on-one interactions with SPs, helping them to provide a safe space and get the pulse on the dynamics of the communities they are serving. As one SP in FG1 emphasized, there is a need for SPs to ensure the voices of the most marginalized are also recognized. For this SP, activism means a priority was “to get out into the community and meet and hear what’s going on” (FG1) in order to understand and represent these diverse community perspectives as evidence to inform directions for developing meaningful programs and services to meet their diverse needs. The nature of SP activism in SP narratives included improving organizational resources for clients to avoid service barriers. As one SP explained, “We have a lot of non-status, uninsured people that require services at the agency where I work. And some of the services they need they are not qualified for… What can I do? I also know there are no services that are serving everyone. So… creating program services for them… [is] a step towards activism” (FG1).

#### 3.2.2. Multilevel Transformative Action

Settlement sector SPs in FG2 considered the nature of their everyday MHW promotion advocacy *with* and *for* racialized immigrant women. Transformative actions to address social and structural dynamics reflect engagement with individuals (micro), organizations (meso) and the larger system (macro). While remarking that they do not use terms such as *mental health or health promotion* in this sector, these SPs shared strengths-based MHW strategies at the individual level (between SP and client) to foster women’s understanding of MHW and self-care and enhance women’s confidence and social support such as peer networks to reduce isolation. Their strategies align with MHW approaches that often take the social determinants of health into account such as social support as well as foster their agency. However, most SPs across FGs pointed to normative structural and system-level dynamics shaping immigration and settlement policies such as lack of recognition of professional credentials and requirements for Canadian work experience that contribute to underemployment and juggling of precarious work for survival in Canadian life. In addition, some SPs spoke up about the blatant immigration and settlement system that continues to deny racialized immigrant women their humanity as they engage in the mire of bureaucracy for years that denies them their citizenship. One SP noted that the agency philosophy can be an important factor in relation to actions that SPs take to challenge oppression, identifying that the agency philosophy can often be grounded in a political ideology that is not helping those in need, making the health inequities continue. A FG1 SP whose agency takes an “anti-colonial approach” shared, “We do have a lot of service users who’ve been in this country for years. Most of them do not have their citizenship because immigration likes to delay their papers” (FG1). As they noted, “We help these individuals complete and send their papers… four, five, or six times. This requires money and time… the issue was not the…. people… [but] the system” (FG1). For SPs who are well aware of the bureaucratic barriers to achieving citizenship that their clients face, such scenarios are the impetus for SPs to take individual and collective action with professional and organizational colleagues at the system level, protesting what they see as unfair practices and systemic inequities that are clearly linked to structures of colonization and racialization. As these SPs suggest, an explicit organizational commitment to addressing these deeply embedded structures facilitates their understanding of effective policy activism.

#### 3.2.3. The Anti-Colonial Approach and Activism

Several SPs working in mental health services agreed that their anti-colonial approach also helps them point to the underlying philosophical issues, such as the biomedical dominance of the immigrant service, including the mental health system, and informs the direction of their activism. For instance, one SP (FG1) stressed the importance of paying attention to making SP’s voices heard about the biomedical framing of mental health, diagnosis and treatment discourses that predominate in Westernized North American health and social systems at the expense of other perspectives and practices. Several consider the implications for the way that the MHW of racialized immigrant women is interpreted by the SPs who have the authority to determine how their MHW is addressed. As one FG1 SP indicates, “Sometimes people try and advocate for service users” but in doing so, actually what can happen, is that approach retraumatizes or stigmatizes the person resisting the system. This SP suggests that, given historical concerns about how the traditional mental health system has not served racialized and/or women well, options to support diverse racialized immigrant women clients’ MHW needs must go beyond the diagnostic and treatment focus of psychiatric and psychological services and mental health system that are anchored in the dominant Westernized biomedical model or risk exacerbating mental distress. For this SP, recognizing that meeting a client’s needs is equated with “not wanting to go back to these services… that in itself is also activism…” (FG1). One SP, whose agency they describe as very politicized, reflects an openness to challenging the prevailing mental health system. They note that “diagnosis… any mental health… agency… can be extremely problematic… very Westernized… a real problem… across the board for a lot of immigrants. It is important to understand that, sometimes not accepting the diagnosis… somebody else’s understanding of MWH, is a form of activism” (FG1).

#### 3.2.4. Counternarrative: Ableism, MHW and Activism

These SPs provide a counternarrative to dominant conceptualizations of client MHW and SP practice strategies as a form of activism that align with resistance to the Westernized biomedical model that prevails. Strategies that can support this counternarrative include taking a postcolonial stance to foster understanding that normative social structures are implicated in processes that stigmatize both ableism and MHW. As one SP points out, whether individuals are seen as capable and thus able-bodied, there are implications for expressing resistance to the prevailing biomedical mental health system that takes ableism into account. This SP considers that prevailing notions of activism are also relevant, saying, “How society frames activism, it can also be very ablest” (FG1), suggesting that dominant notions of activism with their focus on mass protests or letter writing to politicians are built on normative assumptions that an able-bodied (i.e., articulate and rational) white middle class citizenry has the freedom to express protest. In the prevailing Westernized biomedical mental health system, deeply embedded practices delegitimize knowledges that are counter to the authoritative and dominant biomedical regime. Such practices, along with treatment regimes, have been critiqued as dehumanizing for individuals assumed to have or who have been diagnosed with a mental health disorder. For racialized migrants, these dynamics echo colonial practices which have created them as “the Other”, erasing or marginalizing their knowledges and practices and limiting their expressions of agency. All who engage with the mental health system contend with stigmatizing stereotypes that link mental health diagnosis to risks for disorderly and violent behaviour. That can have implications for authorities in the medical and justice system to regulate their freedom, enforcing restrictions on their behaviour and thus their ability to participate as full citizens in public spaces. Many racialized immigrant women, who are already perceiving that bureaucratic immigration and settlement systems deny them their humanity and citizenship, with good reason fear engaging with the mental health system which can actually put their ability to achieve Canadian citizenship at risk. Taking these complex oppressive system dynamics into account is important context for SPs who engage with racialized immigrant women. This SP then links whether an individual is perceived as or deemed to have a mental health disorder to the legitimacy of their expressions of activism: “So, … if somebody is screaming on the street, to you, that may not be activism. If somebody’s on the street corner, that may be, it’s like ‘anti-psychiatry.’ It’s freeing: ‘Yes, I am mad, and deal with it!’” (FG1). They stress that anti-psychiatry is “something big that’s coming up now that a lot of people care about” (FG1). The naming of colonialism and ableism as relevant to racialized immigrant women’s MHW support and SP MHW practices aligns with a broader holistic view of MHW support systems that foster client agency and activism that accounts for how intersections of ableism, colonialism and racialization influence their experiences of MHW care. Another SP in the settlement sector points to implications for both SPs and clients when their interaction to promote MWH is not based on a psychiatric label. “I don’t want to look at you and think of this negative title that you’re carrying around like an unnecessary piece of clothing that you want to cast off from yourself. It can be very stigmatizing” (FG1).

#### 3.2.5. Investing in Activism: Weighing the Personal and Professional Considerations

As SPs shared often passionate stories about how they demonstrated resilience and resistance to workplace challenges through activist practices to push against oppressive systems, they also shared insights into personal and professional considerations that contextualized the nature of their activist commitments with potential implications for their MHW. Their practices to prioritize community collaborations at times were outside of the scheduled work hours and that required significant personal resources. While one SP shared, “For me, advocacy is changing my way of working according to the constraints of my agency,” another SP responded, saying they “don’t just advocate, but actively fight for an important program by bringing groups with common concerns together” (FG1). Community organizing is a form of activism that goes beyond writing letters to policy makers. To make it impactful, FG1 SPs agreed that extending their actions to involve communities often requires considerable work on weekends and evenings, using their personal resources, time, and money. One SP explained how they put enormous amounts of personal time to “fight for the program… very close to my heart… [The program] was causing them a lot of family breakdown, mental breakdown…. because of the [lengthy] processing of their papers…” (FG1); they eventually saw good outcomes in this case.

But, as several SPs note, there can be work- and health-related consequences such as job security related risks for SPs who engage in this activism. As one SP indicates, “There’s… a common call for a change, but… sometimes is it’s hard to go against that… we worry about our jobs and positions… It’s very, very challenging, especially when you see something that needs to be improved” (FG1). They then agreed with one of the FG facilitators, who summarized this, saying, “Sometimes you are even afraid… for your position to be eliminated because of your activism work” (FG1). One SP stressed how these activities are related to MHW, saying, that it could be much more stressful and traumatizing, “if I stay quiet… [it] may lead me to depression… [and anger]” (FG1). Despite the risks, however, many of these SPs are involved in activism, breaking the silence on the conditions that impact their own MHW and the structural and social conditions that contribute to long term moral distress and vicarious trauma that shape their ability to act.

## 4. Discussion

By centring the voices of these SPs, many of whom were racialized immigrant women themselves, and using a postcolonial feminist analytic lens, the FG findings unexpectedly uncovered themes of SP vicarious trauma and burnout, as well as SP activism, and impacts of this MHW promotion practice on SPs’ own MHW. We now contextualize our findings in the literature, illuminating the complexity of SP experiences and activism and counternarratives about SP MHW emerging with a postcolonial feminist lens.

### 4.1. Trauma and Violence-Informed Care

These findings align with the literature on trauma- and violence-informed care that not only addresses the need to create safe spaces for all of their clients and avoid retraumatization but also considers SP needs, e.g., [28,30,40,64,65,66,67,68]. In particular, this study contributes to an understanding of the MHW risks for SPs working with immigrant and refugee women survivors of trauma and the relevance of structural dynamics of gender, racialization and colonialism in relation to creating a supportive organizational climate for SPs. At times, our FGs highlighted how client stories resonate with SPs’ own lived experiences of trauma and settlement as racialized women and contributing to vicarious trauma. Our SPs described everyday practice whereby tight client scheduling and precarious settlement programming constrained available client supports with impacts on SP MHW. SP moral distress is exacerbated by many SPs’ perceived inability to speak out about the trauma and overwhelming workplace stress they experience that undermines healthy working conditions [13,37]. A workplace “climate that is itself trauma-informed” [40] (p. 81) and attends to structural violence requires attention to policies and self-care practices that foster safety for both SPs and clients [28,40,41,42,66]. Our findings align with approaches that anticipate that SPs can experience indirect trauma and recommend that organizations take responsibility for developing a work environment “that validates and normalizes workers’ reactions and mitigates [SP MHW] risk… in ameliorating indirect trauma… as much as an individual [responsibility]” [40] (p. 81).

Dewey et al. [42] noted that “COVID-19… upended clinicians’ sense of order and control” (p. 752). However, our findings support other literature which suggests that SPs’ pre-COVID working conditions and experiences were being shaped by experiences of trauma, emotional upheaval and moral distress related to working conditions, uncertainty, and frequently changing policies [15,27,44,45,48]. In order to represent meaningful commitments to equity, rather than individual behavioural solutions for SPs, principles of trauma and violence-informed care, which account for structural dynamics affecting various sectors and diversely situated SPs are recommended. Settlement and mental health are female-dominated health service sectors that historically and continuing today are often dominated by racialized and/or female SPs [4,13,35,36,38]. Our findings suggest that our SPs, who for the most part are racialized women and have Canadian immigrant experience, often feel smothered in the system. Many do not want to risk losing their jobs by speaking out. So, while they are very resilient and want to be “good citizens,” these tensions contribute to their silencing and burnout. At the same time, they advocate on behalf of clients and engage in activism to the extent possible.

### 4.2. Activism and Agency Response

These FGs provided a space for these highly committed SPs to break this silence on the vicarious trauma and experiences of burnout that they face, sharing their concerns; instead, when shared one-by-one, these concerns might be attributed to their individual failings, their emotional makeup or their personal characteristics, as has been characterized as problematic when interpreted through an individual, biomedical framing of women’s MHW that dominates [39]. Despite their diversity of background and experience, SP thoughtful analysis points to the complex micro (individual), meso (organizational) and macro (system) dynamics that shape their lived experience of practice [15]. In this study, conducted several years prior to the onset of COVID-19, SPs unexpectedly shared their everyday experiences of moral distress, burnout, and vicarious trauma, along with emotional upheaval that they attribute to organizational and system-level issues which make it challenging to meet client needs, whether they are working in mental health or settlement services. Yet, SPs’ silence, perceived challenges of speaking out in their organization about these factors, suggest that they are subjugated knowledges, aligned with what has been described as structural violence [13,25,27,39]. Our findings resonate with critical research undertaken pre- and post-pandemic that calls for activism at multiple levels and anti-oppression strategies that enhance resilience and capacity building and that are also aligned with trauma- and violence-informed care and cultural safety to support SPs that address structural violence shaped by gender, racialization, colonialism, neoliberalism, and their intersections [7,13,27,28,34].

Our findings revealed how intersectionality, based on multiple social identities, such as race, gender, migration status, sexual orientation, and others, intersects to create unique experiences of privilege and oppression among SPs. Along with racialization, gendered norms, which include heteronormativity and cisnormativity, must also be considered as relevant to the everyday practice dynamics that our SPs face and the factors that shape their actions [22,23,30]. Our study also illustrates how gender and racialization and their intersections with other normative dynamics are relevant to the nature of SP work with racialized immigrant women, many of whom in immigrant service agencies are themselves racialized women, and in our study, also those who have Canadian experience of migration. Consistent with Gailits [59]’s study with Latin American, female immigrants, these SPs are all too aware of what she describes as “migratory distress” experienced by migrant women as they “hold on” “being strong” and “silent,” navigating dashed expectations of Canadian life. Instead, their everyday lives are shaped by stress related to lack of permanent residency and the precarious work that they juggle and exacerbated for many by loss of close community. Characterizing these experiences as being “stuck in a highwire act” Gailits [59] explains the structures of colonization, patriarchy and racialization that shape the context of migrants’ lives, the epistemic violence they face, and the importance of critical mental health approaches that go beyond biomedical understandings to meet their needs—as well as providing a context for understanding how they exercise agency to challenge the oppressive circumstances they face.

Although our SPs did not explicitly articulate phrasing of “race-based trauma,” features of their activism extend findings from the literature on race-induced trauma in the critical education literature on Black women’s experience [67] and discrimination in the psychological trauma literature [68]. The global Black Lives Matter movement and COVID-19 pandemic have sparked concerted attention to racialization as a factor in health inequities for SPs in a Canadian context that has focused on Black, Indigenous and to some extent Asian women [1,2,3,8,28,69]. Our use of a broad definition of racialization in our study that considers women “who are subject to racism and made to feel different because of their race, skin colour, ethnic background, accent, culture or religion” [29] (p. 1) extends the literature on SP MHW related to workplaces, activism, trauma, discrimination, e.g., [2,3,8,13,22,23,28,30,36,37,49].

SPs in our study, like Singh’s [36], who were SPs working in ethno-specific anti-violence agencies for women and Ku’s [35] immigrant-service NGOs in Toronto, Canada, spoke of the challenges that they face working in the female-dominated human service sector. Racialized minority immigrant women SPs in these studies explained how neoliberal discourses of efficiencies that shape their workplaces, along with the intersection of gender, colonialism and racialization are relevant to their practice as SPs. However, they creatively worked within and outside of their agencies to better serve their clients by addressing underlying structures implicated in systems that contributed to racialized and gendered violence (pp. 509–510) and also contributed to SP moral distress. Our findings also resonate with Ku’s [35,37] research with racialized minority immigrant women who are activist managers in immigrant-service NGO. Although participants in our study were not identified as holding managerial positions, their FG narratives strongly reflect SP actions and community commitments that ultimately put a priority on whether their clients are ‘served’ [37] (p. 74). As with Ku’s [37] activist managers who are also well educated, often holding professional and/or graduate credentials, our SPs, who at times refer to anti-colonial and/or disability theory in their narratives, rather than “intellectualizing,” demonstrate a focus on being seen as a “doer” as tackling individual, organizational and system-level problems. They “[claim] a voice of difference where one’s practice or experience is foundational in claiming insider knowledge” [37] (p. 74) that gives credibility to their activism with the communities. “Personal struggles of marginalization as well as the manifestations of structural violence service providers witnessed in the course of their work and activism” [36] (p. 509) contextualize racialized SPs’ activism working in ethnic-specific agencies [36,38]. Singh [36] stresses the multidirectional nature of knowledges and practices “that activists derive through their professional lives ‘here and there’” [36] (p. 506) that affirm the complexity of their understanding of their power and privilege as activists and racialized women in both the current North American work context and prior to migrating. Using a postcolonial feminist lens, Singh’s [36] findings with Asian SPs in effect, act as a counter discourse to dominant Western notions that characterize and uphold binary notions of the “orient” as backward and barbaric, and West as modern and progressive.

While “breaking the silence” on their own MHW experiences, SPs share that they often feel compelled to undertake action to transform the system to rectify concerns and be more responsive to diverse racialized communities’ needs. In doing so, they illustrate how they are actively engaging in activist practices themselves, responding in transformative ways to challenges in the current practice environment, see [28]. Their activism often aims to bolster social change that can have impacts on improving the everyday lives of their clients through enhanced programs and resources. Yet, the career and job risks they take as they speak out and act must be contextualized in relation to the female-dominated health and social services sectors. Rather than a “generic” consideration of self-care, as they push against the system, their actions are consistent with what Waite and Iherduru-Anderson [69] describe in relation to racialized nurses who must speak out and act: this “radical self care” (p. 3) is “good trouble” (p. 3). These FGs capture collective SP voices and point not just to their experiences of serving clients on a one-to-one basis, but the actions they take, consistent with activism to challenge the system. As they speak, they identify the deeply embedded structures that inform both their personal and professional lives and those of the racialized immigrant women they serve. Since the prevailing notion of self-care in a MHW context is about individuals taking care of themselves as individuals, the counternarrative of radical self-care which is about actions to enhance collective wellbeing to address racism aligns with the actions of these SPs to address deeper structural dynamics and thus trauma- and violence-informed care [29].

### 4.3. Counternarratives on MHW and Practice

Study findings reflected critical MHW counternarratives at both individual and organization levels that challenged prevailing biomedical and Westernized approaches. SPs questioned the stigmatizing psychiatric labelling that aligns with the dominant Eurocentric mental health discourses as key to promoting racialized immigrant women client MHW [39]. Such findings also aligned with literature that foregrounds structural racism in undermining racialized women’s perspectives and experiences as relevant to MHW [2,3,9,34]. Less visible in health services or nursing research, even that informed by postcolonial feminism, is explicit attention to SP MHW identifying the intersections of neoliberalism, gender and racialization with ableism and activism as relevant to the structural violence shaping MHW, e.g., [28,39,70]. Yet, as Liegghio [56] explains, non-recognition of “psychiatrized people as legitimate knowers with legitimate knowledge and ways of being in the form of epistemic violence constitutes a major form of injustice for people labelled with mental illness” (p. 129).

Finally, in the context of SPs’ complex personal and work-related experiences, SPs also shared key ideas that speak of their resilience and self-determination [28,37]. However, there appear to be few studies that account for the complexity of dynamics that shape SP MHW as they express their agency through political activism. SPs’ strengths and strategies to work within and outside of the system are shaped by factors such as their understanding of the system, their perspectives advocating for racialized populations, and their personal experiences as racialized immigrant women. In our study, despite the work-related challenges, SPs themselves demonstrate strength and self-determination that aligns with what Knight [40] describes as ’vicarious resilience’…. that can include “a re-ordering of personal goals and priorities, increased sense of professional competence and resourcefulness, and heightened capacity for compassion and empathy” (p. 81), see also [28].

These findings highlight how these SPs take into account the dynamics of the authoritative Westernized and biomedically focused mental health systems that dominate [55,70]. They identify normative assumptions about activism that overlook ableism as a factor that must be considered in relation to MHW. Ableism also has implications for whose voices can be heard to transform systems of care, approaches congruent with critical mental health research that foregrounds survivor activism [39,52,53,70]. Practices to advance health equity for diverse groups of people who have been marginalized or harmed by dominant systems of MHW care, align with McGibbon et al. [25] who stress the importance of understanding the complexity of structural factors that undermine equity.

This nursing research aligns with critical social science research including critical feminist work based in social work and other health professions, and more recently nursing [39,51,52,53,71,72,73], that takes issue with the authoritative biomedical underpinnings of psychiatry. These researchers problematize the epistemological roots of psychiatry (based in eugenics, for instance), highlighting the lack of evidence base for diagnostic labelling that has shown disproportionate harms that racialized, gendered and other disenfranchised communities can face. Our study, which foregrounds SP voices, the complexity of structural dynamics including colonialism, racialization and gender shaping SP MHW transformative narratives of SP activism, make visible silences, subjugated knowledges and counternarratives that challenge the legitimacy and limitations of prevailing biopsychiatric Western understandings and practices of MHW promotion. Our findings provide a counternarrative to generic understandings of activism and activist practices in a nursing context. Such research aligns with Rosario et al.’s [62] recent scoping review to examine the nature of calls for “decolonizing nursing.” There are implications for using a postcolonial feminist lens to identify meaningful and comprehensive anti-oppression strategies that take colonialism, racialization and gender into account to decolonize nursing practices. Fostering health equity for diverse racialized women requires concerted attention and multilevel strategies informed by anti-oppression, e.g., [1,3,9,15,24,34] and aligned with critical mental health promotion practice and trauma- and violence-informed care.

### 4.4. Limitations

The CBR approach in which racialized immigrant women communities themselves were actively engaged across the research was a strength of this research. This was a small, purposive convenience sample of 19 SPs. However, the study participants may have been limited to networks associated with the research team and community partner in the GTA. The nature of focus group methodology may have limited recruitment given the potential for discussion of sensitive topics such workplace resources, experiences of trauma and lack of workplace support as well as potential identification to communities and SP availability to participate given their workloads. The majority of SP participants self-identified either in the demographic questions or narratives that they had experience as racialized immigrant women in a Canadian context. Data collection occurred prior to the COVID-19 pandemic. While this is useful, further research using similar qualitative methodologies, mixed methods and implementation research is needed to understand the needs and supports for SPs across sectors and across geographic locations beyond the Canadian context.

## 5. Conclusions

Through this Canadian CBR with racialized immigrant women, we aimed to build SPs’ capacity to support strengths-based MHW promotion approaches such as activism to meaningfully promote client MHW. We applied postcolonial feminist and critical mental health promotion lenses [32,34] to understand the personal and professional impacts of this practice on SPs themselves and strategies to support them. The findings offer insight into the nature of the added value of our research to the existing research on trauma- and violence-informed care. Using a postcolonial feminist lens draws attention to insights informed by centring diverse SP voices and making visible their subjugated knowledges, their strength and agency and the complex structural dynamics implicated in promoting the MHW of diverse racialized immigrant women. Using both postcolonial feminist and critical MH promotion lenses we contribute to the emerging body of literature that challenges the relevance of the dominant and Western biomedical lens in MHW promotion as it relates to gender, racialization and colonization, expanding the critical literature on racialized immigrant women SPs. These findings highlight the structural underpinnings of the intersecting dynamics of gender, colonialism and racism that contribute to the vicarious trauma and burnout that are impacts for SP who promote MHW of racialized immigrant women.

The findings point to recommendations for critical health and social research and nursing practice that call for comprehensive strategies that explicitly take into account gender, colonialism and racism for a meaningful and supportive organizational climate about trauma- and violence-informed care and MHW promotion for racialized and/or immigrant SPs. Our findings affirm how diverse SPs work within and beyond the system using strategies aligned with activism to promote their own MHW and ultimately their clients’. Our findings also draw attention to ableist discourses that co-exist with and operate in conjunction with racialization processes and forms of epistemic violence that undermine what activism looks like and who has the legitimate authority to participate—and provide a counternarrative to generic understandings of activism and activist practices in a nursing context. We argue that the findings offer insights into directions for decolonizing nursing knowledge and practice in relation to MHW promotion that align with health equity goals, especially in a nursing context [25,62]. They point to the comprehensiveness of strategies that are needed to decolonize nursing at the intersection of colonization, racialization, gender, neoliberalism with implications for anti-oppressive mental health promotion practice.

## Figures and Tables

**Table 1:** Sociodemographic Information.

Number of Focus Groups and Participants	First Focus Group = 7 Second Focus Group = 6 Third Focus Group = 6 Total = 19 Participants
How did you define your gender?	Female = 18 Male = 0 Other = 1 (Gender Fluid)
Age	Under 30 = 3 30−50 = 13 Over 50 = 3
Highest educational level achieved	Diploma = 1 Undergraduate Degree = 7 Graduate Degree = 7 Professional Credentials = 4
Current type of agency (settlement/mental health/mental health promotion services/mental health & settlement)	Settlement = 7 Mental health = 4 Mental health promotion services = 6 Mental health and settlement = 2
Length of time working in current agency	Less than 5 years = 7 5−10 years = 9 More than 10 years = 3
Length of time working in previous agency	Less than 5 years = 12 5−10 years = 2 More than 10 years = 5
Self-identification as a member of a racialized community (yes/no)	Yes = 14 No = 5
Reported self-identification of racialized community	Chinese = 1 Latin American = 3 Filipino = 1 Latino = 3 Caribbean = 1 South Asian = 2 Middle Eastern = 1 Not identified = 7

## Data Availability

The original contributions presented in this study are included in the article/Supplementary Materials. Further inquiries can be directed to the corresponding author.

## Supplementary Materials

The following supporting information can be downloaded at https://www.mdpi.com/article/10.3390/ijerph22081229/s1, Table S1: Thematic Structure-Example from Selected Theme, Subthemes and Codes.
