# Supplementary material for: Trauma and Activism: Using a Postcolonial Feminist Lens to Understand the Experiences of Service Providers Who Support Racialized Immigrant Women’s Mental Health and Wellbeing
Source: Int J Environ Res Public Health. 2025 Aug 7;22(8):1229. doi: 10.3390/ijerph22081229 (PMC12386781; doi:10.3390/ijerph22081229)
Supplement: Supplementary file 1 [file ijerph-22-01229-s001.zip › ijerph-3701154-supplementary.pdf]

Supplementary Materials

Table S1. Thematic Structure -Example from Selected Theme, Subthemes and Codes.

Major theme 3.1:	Vicarious trauma and burnout		
Subtheme	Subtheme	Subtheme	Subtheme
Constrained client support system	Sector-specific SP training, organizational mandates	Relevance of racialized immigrant women SPs' lived experiences of migration and settlement to their everyday MHW work	SP insights into strategies to support SP MHW
Code	Code	Code	Code
Constrained client support system	Credentials/training linked to sector-specific mandates	Racialized immigrant women SPs	MHW training focus: burnout, moral distress, vicarious trauma
Burnout	Settlement SPs-limited MHW training/connections to resources	Facing/witnessing repeated client stories of trauma	Explicit focus on training/support re: racialized immigrant SP traumas
Limited/changing client resources, programs, policies	Organizational targets	Resonate with own lived experiences of trauma, racism, discrimination	Impact of diverse SP lived experience of vicarious trauma, burnout offers insight into directions for MHW support
Moral distress	Mental health SPs-limited info/resources on settlement/migration	"First responder"	Mechanisms for support: Insights from SPs in teams
Structural influences e.g. gender	Settlement services-safe place for client disclosure/SPs	Settlement services-not stigmatized, safe place for client disclosure/SPs	Silencing-professional stance prevented sharing with co-workers
Structural influences: racialization	"First responder"	SP need for safe workplace	Micro/meso/macro
Social determinants of health e.g., housing	Silos between sectors	Narratives aligned with vicarious trauma	Mental health SP need info on current migration/settlement resources/policies/programs
Emotional upheaval, guilt, overwhelmed/info overload	Clinical credentials/expertise for mental health SPs	Organizational mandates	Peer networks
Heavy caseloads/agency structure	Micro/meso/macro	Personal coping strategies (micro)	Interpersonal, emotional support
Micro/meso/macro	Mandates for settlement and mental health agencies	Increased numbers of clients with mental health crisis	Bridging silos: SP collaborations to develop organizational/system MHW strategies
Relational skills	Stigma of mental health services e. g. (family doctor)	Micro/meso/macro	SP voice
Tensions of practice: Meeting clients where they are	"We are not clinicians" (Settlement SPs)	Structural influences: racialization	Settlement-specific training-access to MHW expertise, referral processes
